# Checklist and Scoring System for the Assessment of Soft Tissue Preservation in CT Examinations of Human Mummies

**DOI:** 10.1371/journal.pone.0133364

**Published:** 2015-08-05

**Authors:** Stephanie Panzer, Mark R. Mc Coy, Wolfgang Hitzl, Dario Piombino-Mascali, Rimantas Jankauskas, Albert R. Zink, Peter Augat

**Affiliations:** 1 Department of Radiology, Trauma Center Murnau, Murnau, Germany; 2 Institute of Biomechanics, Trauma Center Murnau, Murnau, Germany, and Paracelsus Medical University, Salzburg, Austria; 3 Division of Neuroradiology, SALK, Gemeinnützige Salzburger Landeskliniken Betriebsgesellschaft mbH, Christian-Doppler-Klinik, Paracelsus Medical University, Salzburg, Austria; 4 Biostatistics, Research office, Paracelsus Medical University, Salzburg, Austria; 5 Department of Anatomy, Histology and Anthropology, Faculty of Medicine, Vilnius University, Vilnius, Lithuania; 6 EURAC-Institute for Mummies and the Iceman, Bolzano, Italy; Oregon State University, UNITED STATES

## Abstract

The purpose of this study was to develop a checklist for standardized assessment of soft tissue preservation in human mummies based on whole-body computed tomography examinations, and to add a scoring system to facilitate quantitative comparison of mummies. Computed tomography examinations of 23 mummies from the Capuchin Catacombs of Palermo, Sicily (17 adults, 6 children; 17 anthropogenically and 6 naturally mummified) and 7 mummies from the crypt of the Dominican Church of the Holy Spirit of Vilnius, Lithuania (5 adults, 2 children; all naturally mummified) were used to develop the checklist following previously published guidelines. The scoring system was developed by assigning equal scores for checkpoints with equivalent quality. The checklist was evaluated by intra- and inter-observer reliability. The finalized checklist was applied to compare the groups of anthropogenically and naturally mummified bodies. The finalized checklist contains 97 checkpoints and was divided into two main categories, “A. Soft Tissues of Head and Musculoskeletal System” and “B. Organs and Organ Systems”, each including various subcategories. The complete checklist had an intra-observer reliability of 98% and an inter-observer reliability of 93%. Statistical comparison revealed significantly higher values in anthropogenically compared to naturally mummified bodies for the total score and for three subcategories. In conclusion, the developed checklist allows for a standardized assessment and documentation of soft tissue preservation in whole-body computed tomography examinations of human mummies. The scoring system facilitates a quantitative comparison of the soft tissue preservation status between single mummies or mummy collections.

## Introduction

Bioarcheology (scientific study of human remains from archeological sites) provides an insight into the biological status, including diseases, of past people [1]. Paleoradiology is the study of bioarcheological materials using modern imaging methods, such as X-ray radiography, computed tomography (CT), magnetic resonance imaging, and micro-CT [2]. The first CT examination of an Egyptian mummy was reported in 1979 [3] and since then CT has developed into the “gold-standard” for human mummy studies because of its high spatial resolution, image contrast, and non-destructive nature [4–8].

In CT examinations of human mummies within the framework of paleopathological studies, the radiologist is responsible for undertaking the examination to the highest possible standard, as well as for the evaluation and post-processing modalities. In regards to evaluation, one major question for the radiologist will be, “how is the mummy preserved?”, and other questions may include, “are there any pathologies?”, “what was the cause of death?”, “are there foreign bodies or any indicators for anthropogenic mummification?” [9–15]. By definition, mummies are human (or animal) remains with preservation of non-bony tissue [5,16]. The preservation status of a skeleton can usually be described relatively clearly with reference to the presence or absence of bones, their location and alignment. However, description of soft tissue preservation is often limited by vague expressions such as “well preserved” or “poorly preserved”, as mentioned by Wittmers et al. [17] and Aufderheide [16]. Increasing interest in the study of human mummies has reached a point where standardization of its many varying features is desirable [17–20]. One of these classifications is the expression of the degree of soft tissue preservation in a mummy [17]. Therefore, Wittmers et al. [17] and Aufderheide [16] developed a soft tissue preservation system based upon macroscopic viewing of the external surface of mummies across various anatomical segments. As CT provides far more information about soft tissues and organs it should be feasible to also employ this technology for the assessment of soft tissue preservation. However, soft tissue preservation is highly heterogeneous among anatomical locations and tissue types [5,8,13,15,16,21–25]. Checklists addressing this heterogeneity would be an appropriate foundation for a CT-based evaluation of soft tissue preservation.

Checklists are used in both medical and non-medical industries as cognitive aids to guide users through accurate task completion. A checklist is typically a list of tasks or criteria arranged in a systematic manner, allowing the user to record the presence or absence of the individual items listed and to ensure that all are considered or completed [26–30].

Therefore, the primary aim of this study was to develop a checklist for standardized evaluation and documentation of soft tissue preservation in human mummies based on whole-body CT examinations. A secondary aim was to add a scoring system to the checklist in order to allow quantitative comparison of the soft tissue preservation status of individual mummies or mummy collections.

## Materials and Methods

### Materials

CT data from whole-body CT examinations of two different mummy collections were used for the development of the checklist and the associated scoring system. Permission for all aspects of studies was granted by the Order of the Capuchin Friars for the Province of Palermo (Document 40/10) and by the Lithuanian Association of Ritual Services (Document IR-10-33).

The first collection derived from the Capuchin Catacombs of Palermo, Sicily, and consisted of 23 mummies (17 adults and 6 children), most of which can be dated to the late 18^th^ to the late 19^th^ centuries AD. Seventeen of these mummies were anthropogenically mummified, and 6 naturally. The differentiation between anthropogenic and natural mummification was based upon external and radiological examinations. Indicators for anthropogenic mummification were externally visible incisions and radiologically detectable placement of foreign materials into the orbits, the nasal and oral cavities as well as filling of the thoracic, abdominal, and rectal cavities with foreign materials and the evidence of intra-arterial embalming substances (own data) [31]. The mummies were examined by a mobile CT scanner (LightSpeed Plus, GE Healthcare, Milwaukee, Wisconsin, USA; mobile installation provided by Alliance Medical, Warwick, UK). Whole-body CT examinations were performed with slice thickness of 1.25 mm, interval of 1.25 mm (and pitch of 0.75) with 120 kV in standard algorithm [31–33].

The second collection derived from the crypt of the Dominican Church of the Holy Spirit of Vilnius, Lithuania, and consisted of 7 mummies (5 adults, 2 children) tentatively dated to the 18^th^ and 19^th^ centuries AD. All bodies were naturally mummified. The mummies were examined by whole-body CT (Mx8000 Dual, Philips, Best, The Netherlands) with slice thickness of 1.3 mm, interval of 1.3 mm, 120 kV in standard algorithm [34–37].

### Methods

#### Checklist

The checklist was developed following the “Guidelines for Developing Evaluation Checklists: The Checklist Development Checklist (CDC)” [29] with modifications in the following steps.

1. **Focus the checklist task:** The task of the checklist was to assess different kinds of soft tissue from different parts of the mummified bodies in CT examinations.
2. **Make a candidate list of checkpoints:** A list of anatomical checkpoints was proposed. These checkpoints consisted of anatomical structures that are considered in the daily radiological routine and were expected to be detectable on whole-body CT examinations of human mummies based on the authors’ paleoradiological experience and the paleoradiological and paleopathological literature [4–15,18–25].
3. **Classify and sort the checkpoints:** The checkpoints were classified and sorted by means of their location and affiliation to the functional systems of the body.
4. **Define categories and determine the order of categories:** Categories were defined according to anatomical dissection methods as described in the reference work for paleopathology [16], studies on atherosclerosis studies in human mummified remains [38] and radiological reference works [39]. The categories and identified checkpoints were organized from cranial to caudal.
5. **Definition of checkpoint rating:** Each checkpoint was rated “1” when at least any part of the anatomical structure was detectable and “0” when the anatomical structure was not detectable at all. In cases of missing body parts or an incomplete CT examination of a mummy a rating of “n” was assigned for the respective missing checkpoints.
6. **Obtain reviews and revisions of the initial checklist:** The initial checklist was applied to CT examinations of all 30 mummies. Thereby, checkpoints were successively modified, added or deleted to achieve recognizability, identifiability, pertinence, and applicability. All checkpoints that fulfilled these criteria were used to build a finalized checklist.
7. **Delineate and format the checklist to serve the intended uses:** The finalized checklist resulting from step 1 to 6 was formatted in the manner of the “Checklist for Formatting Checklists” [30]. All checkpoints that must be passed were indicated.
8. **Apply and disseminate the checklist:** The finalized checklist was applied to all 30 whole-body CT examinations of the Sicilian and Lithuanian mummies by the first author who is a senior radiologist with 15 years of experience in clinical radiology and 13 years in paleoradiology to obtain data for an example of use.

For estimation of the intra-observer reliability, the checklist was applied to ten randomly selected CT examinations out of the total of 30 CT examinations by the first author three to four months after the first application. For estimation of the inter-observer reliability the checklist was applied to the same ten CT examinations by one of the co-authors who is a neuroradiologist with 30 years of experience in clinical radiology and 10 years of experience in paleoradiology.

Radiological evaluation and performance of multi-planar reconstructions (MPRs) were carried out using the Picture Archiving and Communicating System ImpaxEE (Agfa HealthCare, Bonn, Germany).

#### Scoring system

To add a scoring system to the checklist the quality rating between categories was determined and a value was assigned to each category and subcategory. Numerical values were also assigned to each checkpoint, whereupon it was intended that the same or an equal value be assigned for checkpoints of equal quality (such as the brain, heart, lungs, and the abdominal organs) and that values be distributed as consistently as possible throughout the different parts of the body.

#### Statistics

Intra-observer reliability for selected subsets of items (e.g. A, A1, A2,…) was computed as follows: one out of 10 CT examinations were fixed and the number of observed agreements between the first and second rating of the same observer was divided by the number of all possible agreements. Mean values and 95% confidence intervals for those means were computed. Inter-observer reliability was assessed by the same method on the total of the same 10 CT examinations by one observer.

For comparison of the group of 17 anthropogenically mummified (Sicilian) bodies with the group of 13 naturally mummified (6 Sicilian and 7 Lithuanian) bodies, data were analyzed for outliers and normality by using probability plots. Two-sided, unpaired Student t-tests compared different subtypes of scores (e.g. A, B, A1, etc.). Data were also tested for variance homogeneity, and if this assumption was not fulfilled then corresponding Welch t-tests were used. Ninety-five percent confidence intervals for mean differences were computed. All statistical analyses in this report were performed by using MATHEMATICA 7.0 (Wolfram Research, Inc., Mathematica, Version 7.0, Champaign, IL) and STATISTICA 10.0 (Hill, T. & Lewicki, P. Statistics: Methods and Applications. StatSoft, Tulsa, OK).

## Results

### Checklist

**Fig 1:**
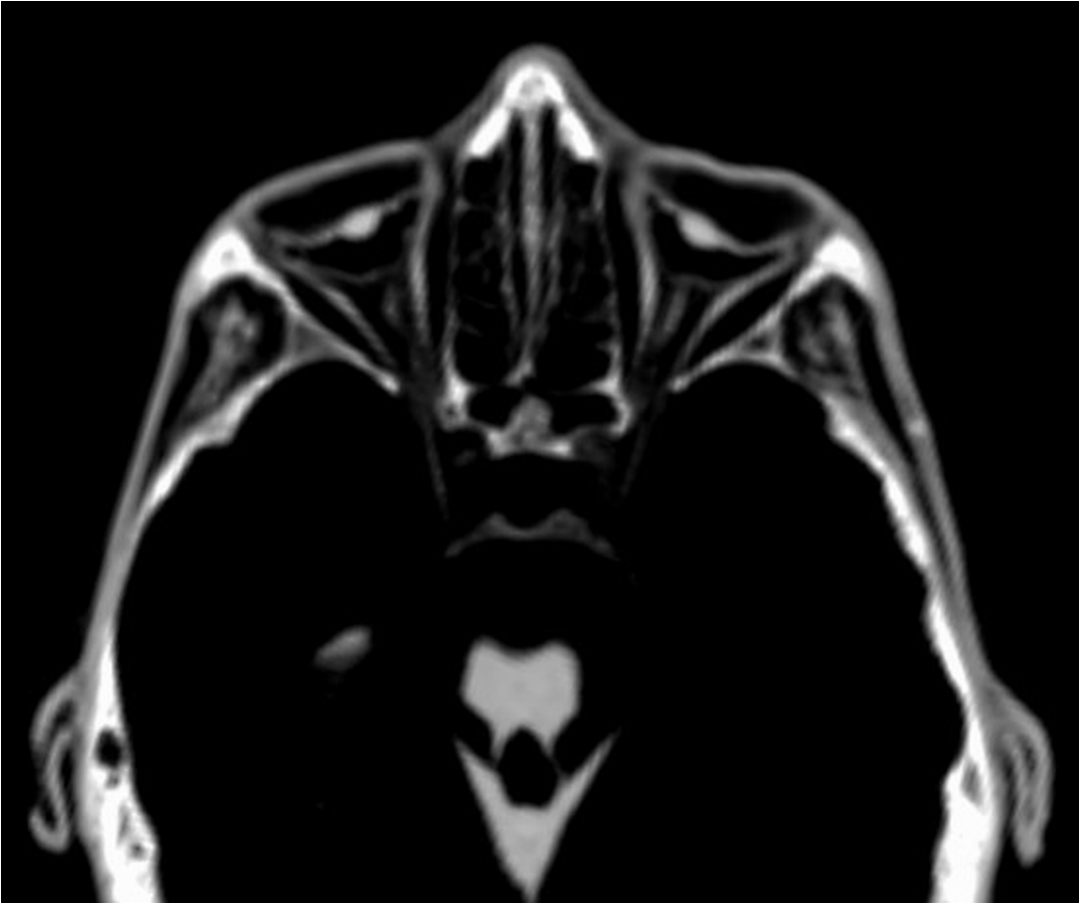
Subcategory Head. Paraaxial multiplanar reconstruction (P11) demonstrates the eye *bulb* bilaterally as an almost semicircular structure. The *lens* is detectable as a small hyperdense structure. The *optic nerve* is visible centrally between the *eye muscles*. The *auricles* are preserved bilaterally. Note *tendons and/or musculature skull-base* bilaterally in the form of the masseter muscle, as well as the *brainstem* and parts of the *cerebellum*.

**Fig 2:**
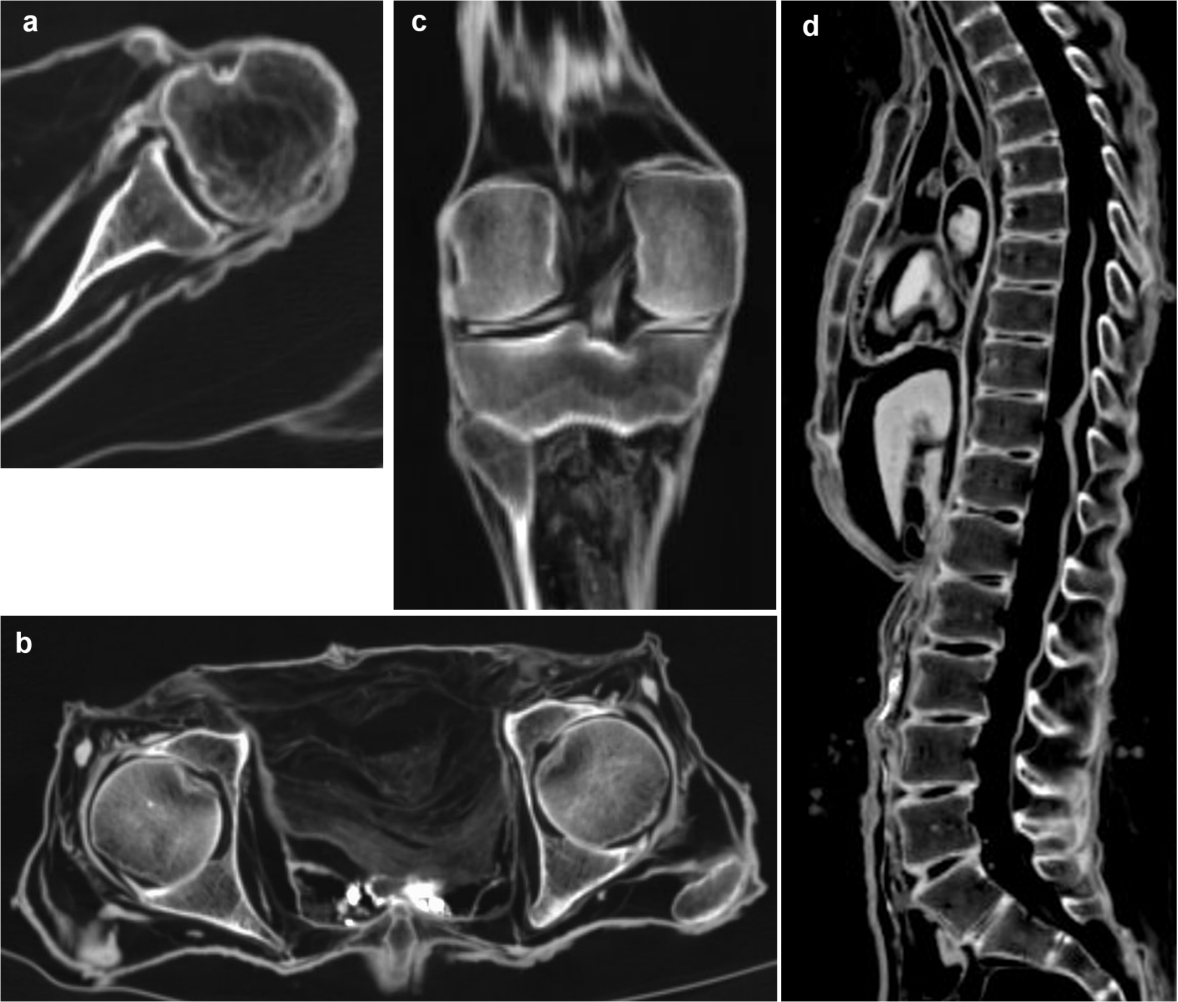
Subcategory Musculoskeletal System. Axial (**a, b**), coronal (**c**) and sagittal (**d**) multiplanar reconstructions. **a** (P1) On the left shoulder the *rotator cuff*, the *long biceps tendon* inside the intertubercular sulcus, as well as the *capsule* and *labrum* are detectable. **b** (P1) Both hips reveal the *capsule* and *labrum*. Note *tendons and/or musculature pelvis*. **c** (P1) On the right knee the *posterior cruciate ligament* and the *medial* and *lateral meniscus* are visible. Note *tendonds and/or musculature thigh* and *lower leg*. **d** (P11) The thoracic and lumbar spine reveals *intervertebral discs* in the form of remnants of the fibrous anulus.

**Fig 3:**
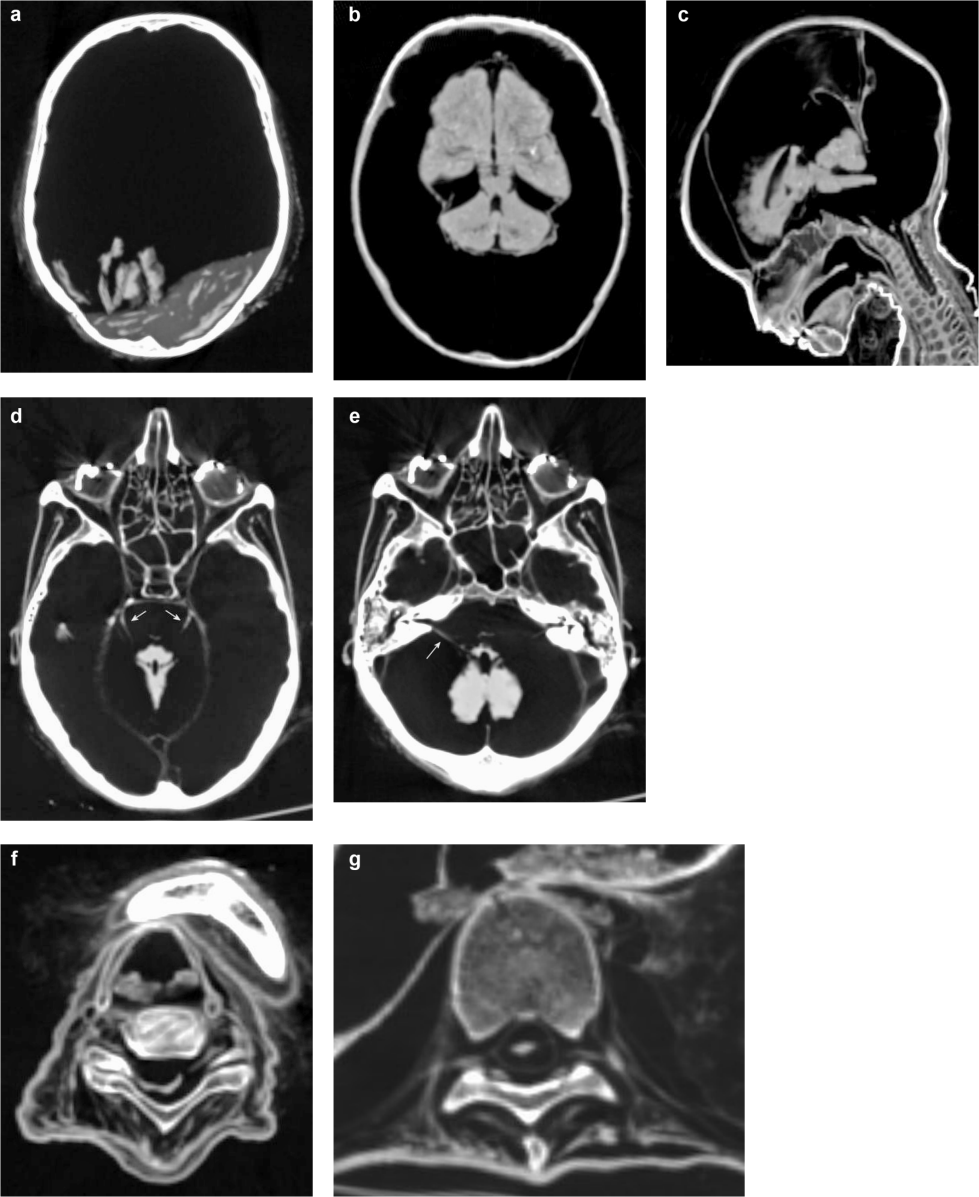
Subcategory Central Nervous System and Peripheral Nerves. Axial (**a, b, d-g**) and sagittal (**c**) multiplanar reconstructions. **a** (P3) Preservation of the brain as a *mass* forming a kind of dried fluid-level at the deepest point of the skull with additional *fragments* of parenchyma. **b, c** (P20) Improved preservation status with identifiable *cerebrum*, *cerebellum* and *brainstem*. Note soft tissues of the *nose*, the *falx* and the *tentorium*, and the *tongue* in **(c)**. **d** (P1) The *trigeminal nerve* (arrows) can be found bilaterally in its anatomical course. Note *bulb and/or lens*, *tentorium*, *falx*, *tendons and/or musculature skull-base*, parts of the *cerebellum*, and *brainstem*. **e** (P1) The *facial nerve* is detectable on the right side (arrow) and suggested on the left side. Note bilateral *auricles*, *bulb and/or lens*, *ossicles*, *tendons and/or musculature skull-base* and *cerebellum*. **f** (P3) The *cervical spinal cord* and *dura* are shown as one single structure. The *peripheral cervical nerves* are visible inside the neuroforamina. Note *tendons and/or musculature neck*, and the thyroid cartilage as part of the *hypophyarynx and/or larynx*. **g** (P1) The *thoracic spinal cord* and *dura* are discernible and the *peripheral thoracic nerves* are detectable inside the neuroforamina. Note *tendons and/or musculature thoracic and lumbar spine*.

**Fig 4:**
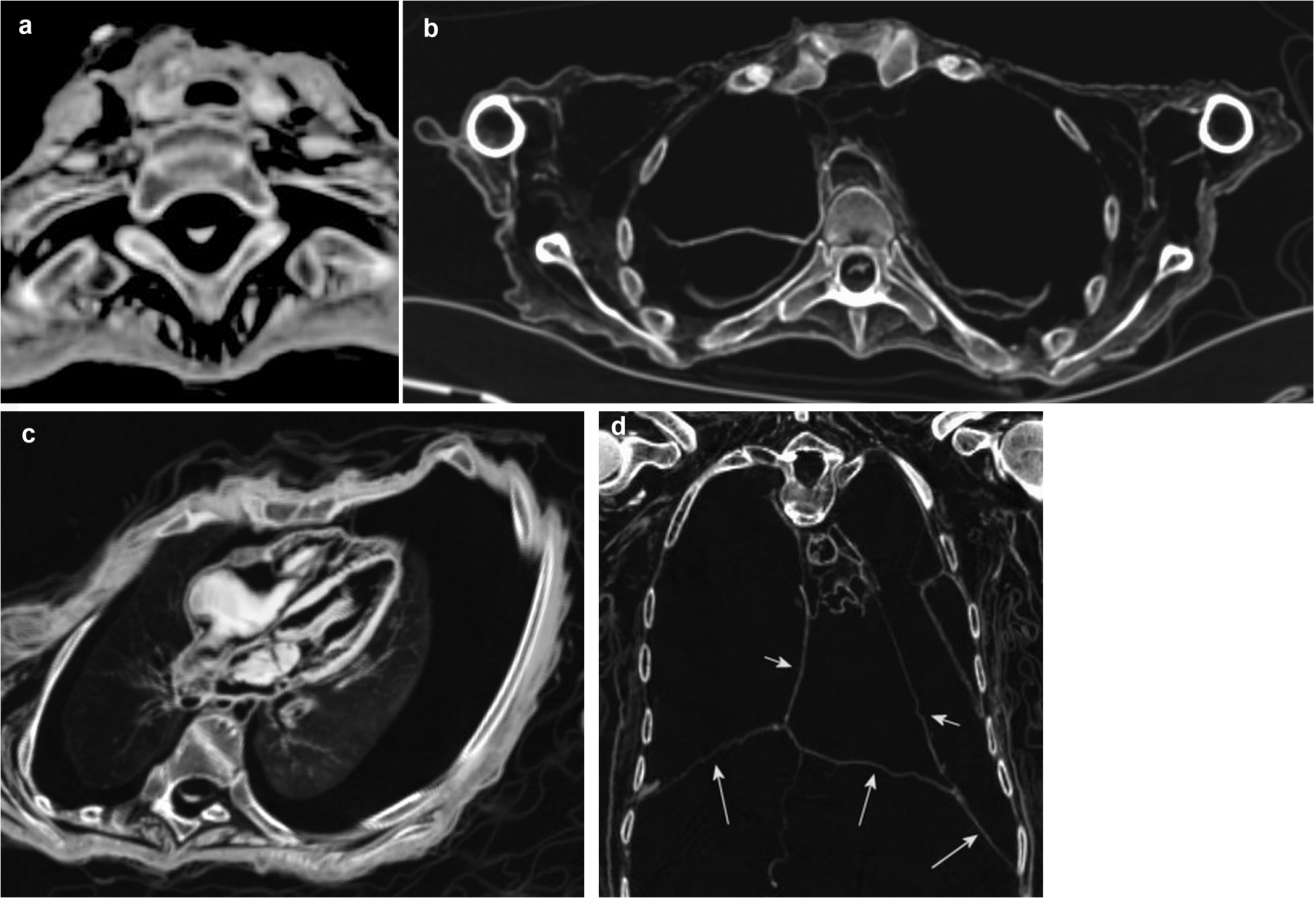
Subcategory Cardiorespiratory System. Axial (**a-c**) and coronal (**d**) multiplanar reconstructions. **a** (P11) The *thyroid gland* is visible as a hyperdense organ surrounding the *trachea*. Note *spinal cord and/or dura thoracic*, *tendons and/or musculature thoracic and lumbar spine*. **b** (V14) Bilaterally, the *lung* is preserved in the form of completely collapsed lobes. Note *trachea*, *spinal cord and/or dura thoracic*, *tendons and/or musculature upper arms*. **c** (P11) Four chamber view of the heart demonstrating *pericardium*, *intraventricular septum*, *four chambers* with filling, *myocardium* and *valves* in a mummy with superior preservation status. Note also excellent preservation of both *lungs*, as well as the *esophagus* and *mediastinal/thoracic arteries*. **d** (P13) Different preservation status of the heart revealing only the *pericardium* (short arrows). The *diaphragm* (long arrows) supports a confident identification of the pericardial sack.

**Fig 5:**
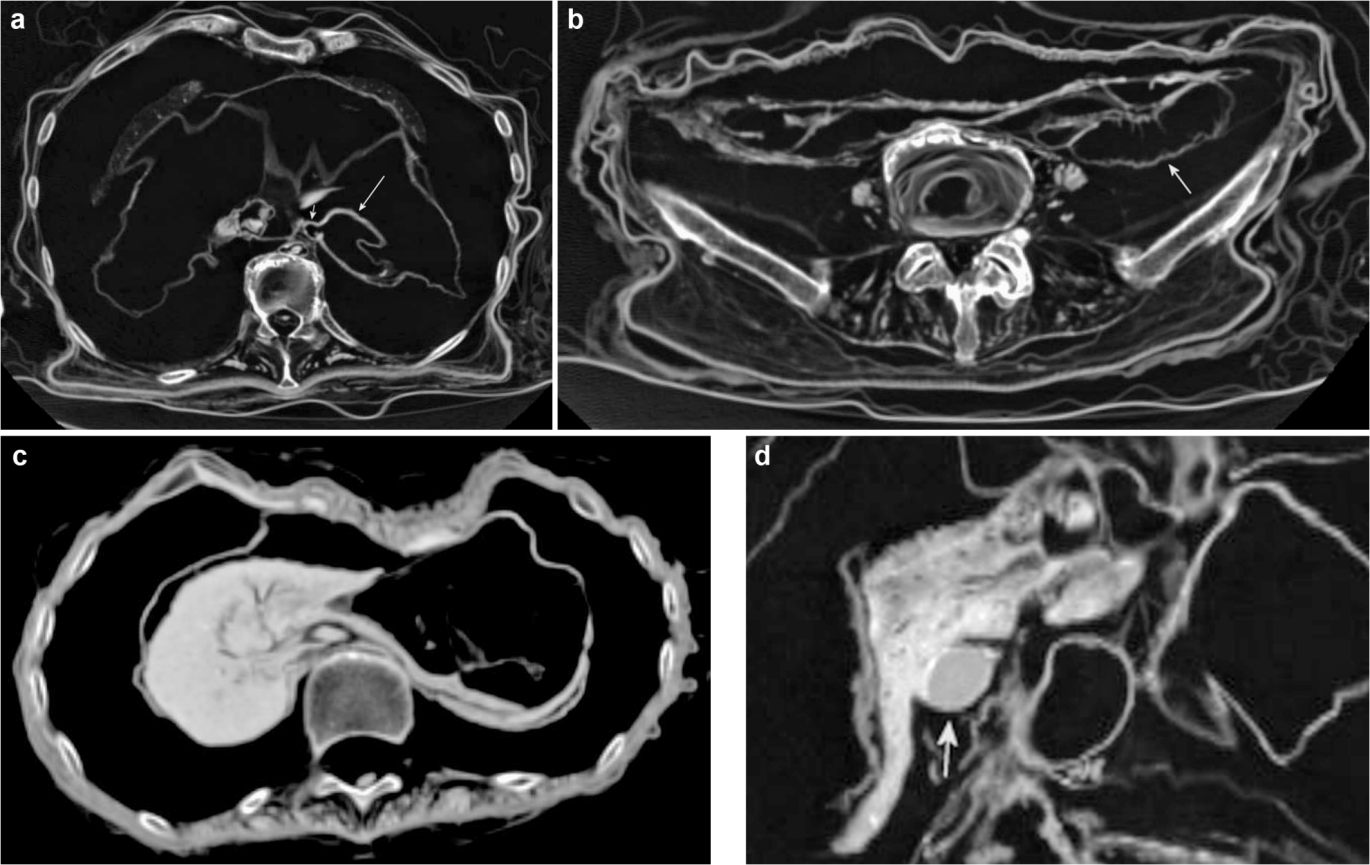
Subcategory Gastrointestinal System. Axial (**a-c**) and coronar (**d**) multiplanar reconstructions. **a** (P21) The distal *esophagus* (short arrow), the passage to the *stomach* and the upper part of the *stomach* (long arrow) are shown. Note proximal *abdominal aorta* with filling, *diaphragm*, *tendons and/or musculature thoracic and lumbar spine*. **b** (P21) The *intestine* is visible in the form of several horizontal layers. In the left abdomen an intestinal loop is clearly detectable (arrow). Note fibrous anulus inside the *lumbar intervertebral disc* space, *tendons and/or musculature thoracic and lumbar spine*. **c** (P11) The shrunken *liver*, *spleen* and *pancreas* are distinguishable. Note strong *diaphragm*. **d** (P21) The *gallbladder* with hyperdense filling and calcifications of the wall is shown in its typical anatomical position (arrow). Note the *liver*.

**Fig 6:**
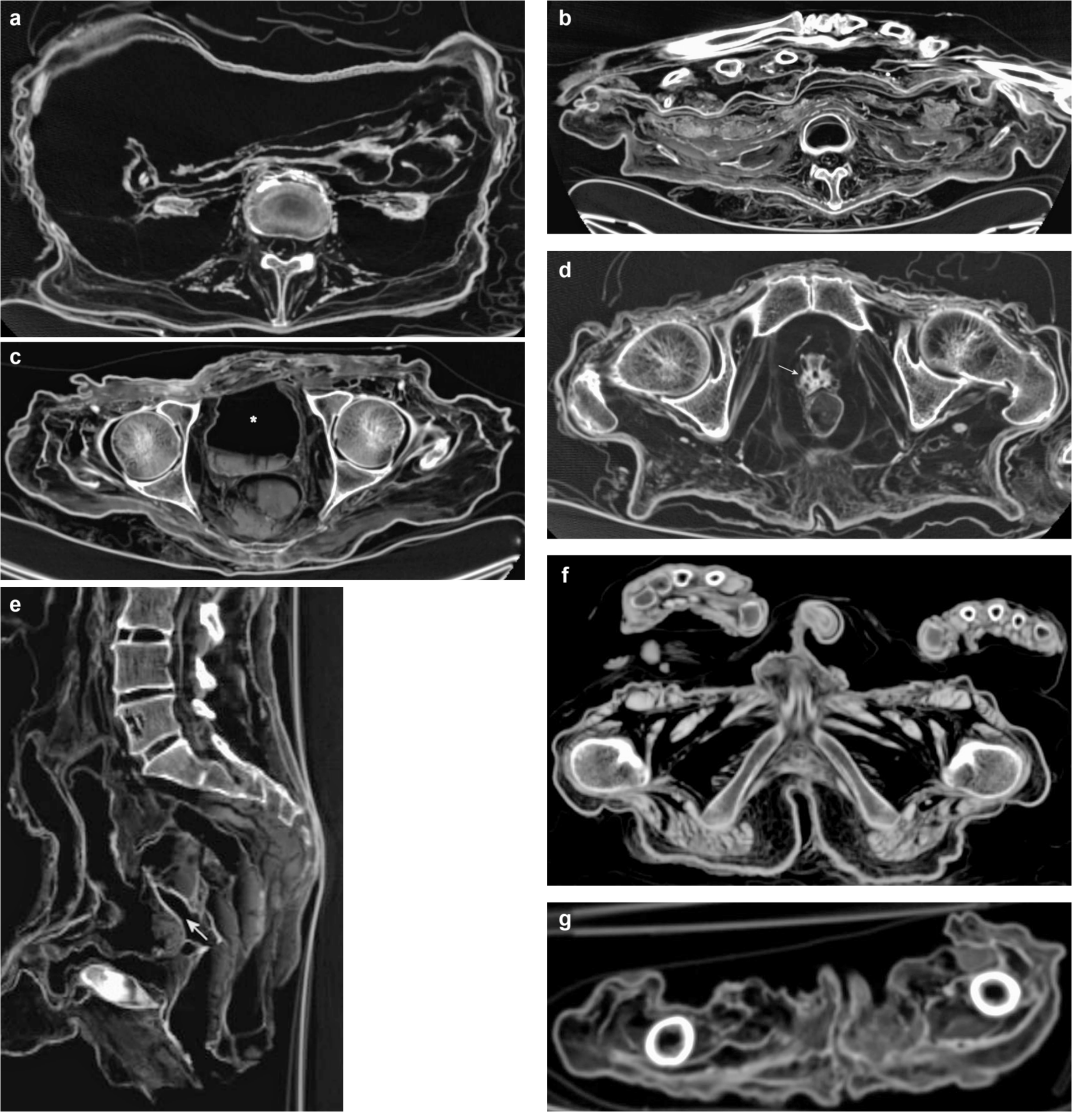
Subcategory Genitourinary System. Axial (**a-d, f, g**) and sagittal (**e**) multiplanar reconstructions. **a** (P21) Both *kidneys* are shown as clearly shrunken hyperdense organs with discernible parenchyma and renal pelvis. Note *peripheral nerves lumbar* bilaterally inside the neuroforamina. **b** (V3) A different kind of preservation of both *kidneys* in the form of hypodense parenchyma and hyperdense margin. Note *tendons and/or musculature right forearm*, *intestine*. **c** (V3) The distended *urinary bladder* (asterisk) is visible with a dried fluid level dorsally. Note *capsule and/or labrum* of both *hips*, *rectum and/or anus* with dried fluid level. **d** (P21) The *prostate* is detectable as a shrunken structure surrounding the urethra (arrow). Note *rectum and/or anus*. **e** (V9) Visible *uterus* with cavum (arrow) and hyperdense margin. Note *intervertebral discs lumbar spine*, *intestine*. **f** (P11) Penis and scrotum are shown. Note *tendons and/or musculature hand*, *tendons and/or musculature pelvis*. **g** (V17) *Labia* are clearly visible in this child mummy. Note *tendons and/or musculature thighs*.

**Fig 7:**
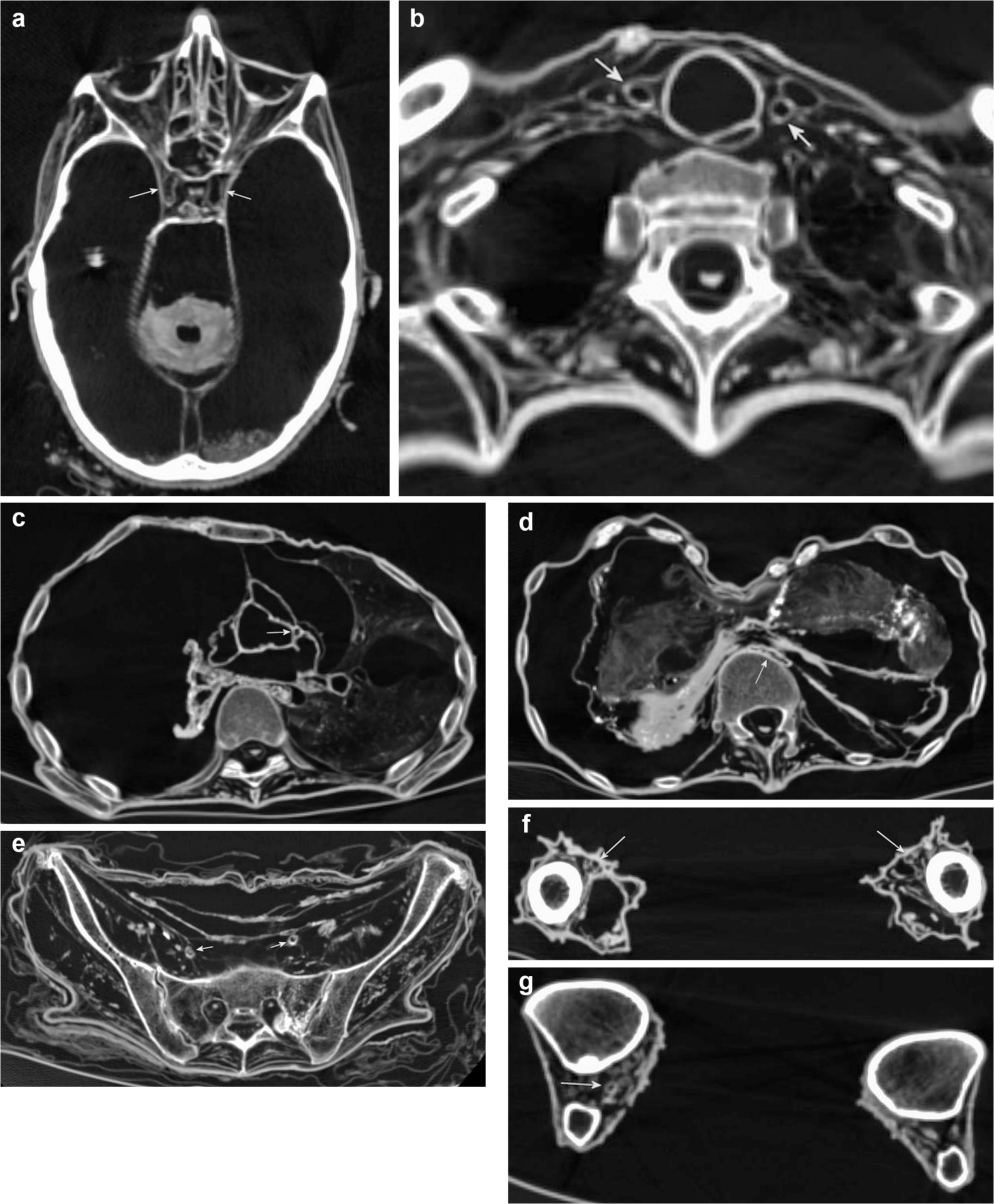
Subcategory Vasculature-Arteries. Axial multiplanar reconstructions (**a-g**) **a** (P21) *Intracranial arteries* are detectable in the form of the intracranial segments of both internal carotid arteries (arrows). Note *bulb and/or lens*, *optic nerve*, *eye muscles*, *auricles*, *cerebellum*. **b** (P1) *The cervical carotid arteries* (short arrows) are bilaterally shown in terms of the common carotid artery. Note *trachea* and *esophagus* dorsally adjacent to the trachea. **c** (P1) From the *coronary arteries* the left descending artery (arrow) is detectable. Note collapsed *lung* on the right side and well preserved *lung* on the left side, as well as *spinal cord and/or dura thoracic*, *mediastinal/thoracic arteries*. **d** (P1) The *abdominal aorta* is visible as a flattened structure prevertebrally (arrow). Note *lung*, *liver*, *spleen*, *tendons and/or musculature left upper arm*. **e** (P21) The *pelvic arteries* are shown bilaterally in terms of the external iliac arteries (arrows). Note *peripheral nerves sacral*. **f** (P1) The *thigh arteries* are bilaterally visible in terms of the superficial femoral arteries (arrows). Note *tendons and/or musculature thigh*. **g** (P1) The *lower leg arteries* are visible on the right side (arrows). Note *tendons and/or musculature lower leg*.

The developed checklist and the scoring system are shown in Table 1. The checklist was divided into two main categories, “A. Soft Tissues of Head and Musculoskeletal System” and “B. Organs and Organ Systems” each including various subcategories, and contains a total of 97 checkpoints (written below in italics). Figures were divided into the subcategories “Head” (Fig 1), “Musculoskeletal System” (Fig 2), “Central Nervous System and Peripheral Nerves” (Fig 3), “Cardiorespiratory System” (Fig 4), “Gastrointestinal System” (Fig 5), “Genitourinary System” (Fig 6), and “Vasculature-Arteries” (Fig 7). Except for the *anterior cruciate ligament* that is comparable to the *posterior cruciate ligament*, and the *tendons and/or musculature feet* that are comparable to *tendons and/or musculature hands*, all checkpoints are shown at least once (Figs 1–7).

**Table 1:** The checklist for assessment and documentation of soft tissue structures in CT examinations of human mummies and the scoring system.

CHECKPOINTS	SCORE	CHECKPOINTS	SCORE
**A. Soft Tissues of Head and Musculoskeletal System**	**100**	**B. Organs and Organ Systems**	**100**
**A.1. Head**	**20**	**B.1. Central Nervous System and Peripheral Nerves**	**10**
*nose*	4	brain *mass/fragments*	1
*auricle* right	2	**or**	
*auricle* left	2	brain *cerebrum*	2
*ossicles* right	1	brain *cerebellum*	1
*ossicles* left	1	brain *brainstem*	1
*bulb and/or lens* right	1	*trigeminal and/or facial nerve*	1
*optic nerve* right	1	*spinal cord and/or dura cervical*	1
*eye muscles* right	1	*spinal cord and/or dura thoracic*	1
*bulb and/or lens* left	1	*peripheral nerves cervical*	1
*optic nerve* left	1	*peripheral nerves thoracic*	1
*eye muscles* left	1	*peripheral nerves lumbar*	0.5
*falx*	2	*peripheral nerves sacral*	0.5
*tentorium*	2	**B.2. Cardiorespiratory System**	**20**
**A.2. Musculoskeletal System**	**80**	*hypopharynx and/or larynx*	1
**A.2.1. Tendons and/or Musculature**	40	*thyroid gland*	5
Neck and Trunk		*trachea*	2
*skull-base*	4	*lung* right	2.5
*neck*	4	*lung* left	2.5
*thoracic and/or lumbar spine*	4	heart *pericardium*	1
*pelvis*	4	heart *intraventricular septum*	1
Extremities		heart *four chambers*	1
*upper arm* right	2	heart *myocardium*	1
*upper arm* left	2	heart *valves*	1
*forearm* right	2	*diaphragm* right	1
*forearm* left	2	*diaphragm* left	1
*hand* right	2	**B.3. Gastrointestinal System**	**40**
*hand* left	2	*tongue*	5
*thigh* right	2	*esophagus*	5
*thigh* left	2	*stomach*	2.5
*lower leg* right	2	*intestine*	5
*lower leg* left	2	*rectum and/or anus*	2.5
*foot* right	2	*liver*	5
*foot* left	2	*gallbladder*	5
**A.2.2. Peri- and Intra-articular Soft Tissues**	32	*spleen*	5
shoulder right *rotator cuff*	2	*pancreas*	5
shoulder right *long biceps tendon*	2	**B.4. Genitourinary System**	**20**
shoulder right *capsule and/or labrum*	2	*kidney* right	2.5
shoulder left *rotator cuff*	2	*kidney* left	2.5
shoulder left *long biceps tendon*	2	*urinary bladder*	5
shoulder left *capsule and/or labrum*	2	*prostate*	5
hip right *capsule and/or labrum*	4	**or**	
hip left *capsule and/or labrum*	4	*uterus*	5
knee right *anterior cruciate ligament*	1.5	external genitals *penis*	2.5
knee right *posterior cruciate ligament*	1.5	external genitals *scrotum*	2.5
knee right *medial meniscus*	1.5	**or**	
knee right *lateral meniscus*	1.5	external genitals *labia*	5
knee left *anterior cruciate ligament*	1.5	**B.5. Vasculature-Arteries**	**10**
knee left *posterior cruciate ligament*	1.5	*intracranial carotid arteries*	1
knee left *medial meniscus*	1.5	*cervical carotid arteries*	1
knee left *lateral meniscus*	1.5	*mediastinal/thoracic arteries*	1
**A.2.3. Intervertebral Discs**	8	*cornoary arteries*	1
*thoracic spine*	4	*abdominal aorta*	1
*lumbar spine*	4	*pelvic arteries*	1
		*thigh arteries* right	1
		*lower leg arteries* right	1
		*thigh arteries* left	1
		*lower leg arteries* left	1

In general, preserved soft tissues were detectable in their approximate anatomical positions as relatively hyper-dense structures with shrinkage of different degrees as described previously [8,9,14,15,40–43]. Checkpoints from category A that mainly represent connective tissue in terms of tendons, ligaments, cartilage, dura mater, and the fibrous anulus of intervertebral discs retained their basic appearance in CT examinations. This was also observed in the mummified bodies as dehydration during the mummification process caused only minor changes in shape and structure. Furthermore, the close relative position of connective tissues to the skeleton allowed identification and classification. In category B it was shown that checkpoints representing parenchymatous organs and hollow organs had clear post-mortem changes observable during CT evaluation. Usually, parenchymatous organs undergo a distinct shrinkage and change of form and structure during the mummification process due to extensive dehydration of the parenchyma [8,9, 14,15,40–43]. Hollow organs were often collapsed and identifiable only as thin membranes. Additionally, the loss of cohesion of soft tissue structures together with gravitational force may have altered the locations of organs.

Further instructions for detection of certain soft tissue structures as well as comments on the definition of certain checkpoints are as follows. *Ossicles*, although representing parts of the skeleton, were assessed bilaterally as possible indicators for preservation of soft tissue, such as the eardrum. Eye *bulb* and *lens* were assessed as one checkpoint, because they were coherent and not distinguishable in many cases (Fig 7A). As the *spinal cord* and the surrounding *dura* were often adherent and not distinguishable, both structures were assessed together, separately for the *cervical* and the *thoracic* spine. Although structures of the *hypopharynx* and *larynx* consist mainly of bone and cartilage they were assessed as a checkpoint indicating general soft tissue preservation of the neck. Calcification of the *thyroid gland* as a result of regressive changes during life should be overlooked as remnant of the thyroid gland, but could help to identify the anatomical region and collateral remnants. Preservation of *lungs* ranged from completely collapsed lungs (Fig 4B) to lungs with widely recognizable parenchyma and vessels (Fig 4C). In cases of pathological change during life, adherence to the chest wall was recognizable. The presence of calcified gallstones or calcifications of the wall of the gallbladder without any further structures of the organ should be discounted as preserved gallbladder. Remnants of *intracranial carotid arteries* were usually detectable as thin septal or tubular structures inside the petrous carotid canal and the anatomical way of the basal intracranial segments (Fig 7A). All other checked arteries were identified as round to oval or flat tubular structures in their known anatomical way (Fig 7B–7G). Calcifications of arteries were helpful for identification, but should be distinguished from preserved arteries.

Application of the checklist to whole-body CT examination of each mummy took between 15 and 30 minutes.

### Scoring system

The maximal scores of the main categories A and B were defined as 100 each. In A, the subcategory “Head” was valued with a maximum score of 20 representing one-fifth, and the subcategory “Musculoskeletal System” with a score of 80. The further subcategories of “Musculoskeletal System” were valued 40 for “Tendons and/or Musculature”, 32 for “Peri-and Intra-articular soft tissues” and 8 for “Intervertebral Discs”. Values assigned to checkpoints ranged from 1 to 4. They were determined to result in the proposed scores for the respective subcategory and at the same time should be distributed as consistently as possible throughout the different parts of the body. In B, the five subcategories were valued predominantly according to the number of included parenchymal organs that were each allotted a maximum of 5 points. Values assigned to checkpoints ranged from 1 to 5. They were determined to result in the proposed scores for the respective subcategory.

### Statistics

The complete checklist had an intra-observer reliability with a mean of 98% (95% confidence interval 97% to 100%) and an inter-observer reliability with a mean of 93% (95% confidence interval 88% to 97%). Detailed results for all subcategories are given in Table 2. By trend, values of the inter-rater variability showed lower agreement in subcategories “B.3. Gastrointestinal System”, “B.4. Genitourinary System”, and “B.5.Vasculature-Arteries”.

**Table 2:** Intra- and inter-observer reliability of the checklist inclusive of subcategories. Reliability is given as a mean (95% confidence interval).

Subcategory	Intra-observer Reliability	Inter-observer Reliability
**All**	98% (97%. . .100%)	93% (88%. . .97%)
**A.1**	98% (96%. . .100%)	97% (93%. . .100%)
**A.2.1**	100%	98% (95%. . .100%)
**A.2.2**	97% (91%. . .100%)	89% (76%. . .100%)
**A.2.3**	97% (89%. . .100%)	97% (89%. . .100%)
**B.1**	100%	97% (92%. . .100%)
**B.2**	99% (96%. . .100%)	96% (90%. . .100%)
**B.3**	98% (95%. . .100%)	87% (78%. . .96%)
**B.4**	100%	89% (81%. . .97%)
**B.5**	96% (89%. . .100%)	85% (72%. . .97%)

The comparison of anthropogenically mummified bodies with naturally mummified bodies revealed higher scores for anthropogenically mummified than naturally mummified bodies for all scores together (p = 0.016) as well as for the main category A (p = 0.008). In A, subcategories “A.1. Head” and “A.2.3. Intervertebral Discs” revealed significantly higher scores (p = 0.002). In category B, “B.1. Central Nervous System and Peripheral Nerves” revealed significantly (p = 0.0001) higher scores (Table 3).

**Table 3:** Differences between the scores of anthropogenically and naturally mummified bodies. Differences are given as a mean (95% confidence interval); n.s.: not significant p>0.05.

Subcategory	Differences	P-value
All	28 (6…51)	0.016
A	13 (4…22)	0.008
A.1	5 (2…9)	0.002
A.2	7 (-1…16)	n.s.
A.2.1	0 (-4…3)	n.s.
A.2.2	4 (-1…10)	n.s.
A.2.3	3 (1…5)	0.002
B	16 (-1…32)	n.s.
B.1	4 (2…5)	0.0001
B.2	1 (-4…5)	n.s.
B.3	6 (-1…13)	n.s.
B.4	3 (-1…7)	n.s.
B.5	2 (-1…4)	n.s.

## Discussion

The developed checklist contains 97 checkpoints proven to achieve recognizability, identifiability and applicability in paleoradiological evaluation of whole-body CT examinations.

The overall reliability of the checklist was high. Differences between intra- and inter-observer reliability were certainly attributable to the careful consideration and experience with the topic by the first observer. Inter-observer reliability showed weaker agreements in the subcategories “Gastrointestinal System” and “Genitourinary System” which contain parenchymatous and hollow organs, with the exception of the tongue. Distinct post-mortem changes of these organs on CT images complicated organ recognition and identification. Difficulties in identification of hollow organs, such as the stomach, were also reported after re-appraisal of radiological examinations of the Tyrolean Iceman [11]. The lowest inter-observer reliability was seen in the subcategory “Vasculature-Arteries”. To assess characteristics of vasculature, arteries were chosen as they have fewer anatomical variants and stronger vessel walls in comparison with veins. However, in the periphery it might be particularly difficult to identify preserved arterial structures due to their small size and difficulty in distinguishing them from adjacent muscular and tendon structures. By trend, reliability was higher in mummies with advanced soft tissue preservation status as it was easier to identify checkpoints.

Some checkpoints of the final checklist were not in accordance with the primary aim because clear recognizability and identifiability of the primary checkpoint did not prove sufficient during the development of the checklist. One important example is the feature “Tendons and/or Musculature”. A major distinction between “tendons” and “musculature” was made. However, there was a continuous transition from clearly detectable preserved musculature to undefined sparse soft tissue structures in the anatomical region of the musculature, which prevented a reasonable classification. Furthermore, the inter-individual variation of the musculature and the possibility of muscular atrophy as a result of immobility or disease before death make this feature quite variable already in its ante-mortem occurrence. In contrast, tendons are easily detectable in CT examinations of mummies as they are anatomically relatively hyper-dense structures [7]. Therefore, we defined the common character “Tendons and/or Musculature” as a subcategory, and given its ubiquity across the body we divided it into 16 anatomical regions.

Despite its small case size for statistical evaluation, the example used showed significant differences between mummification types. These differences indicate that the embalming methods used for the anthropogenically mummified Sicilian mummies [14,31,32] produced an especially high preservation status of soft tissues of the head and spine including external as well as internal structures of the head, and soft tissue structures of the musculoskeletal system as well as peripheral nerves of the spine. Therefore, the preservation status seemed to depend more upon the topography rather than upon the kind of soft tissue in these cases. For application of the checklist and scoring system on single mummies or small mummy collections, a direct comparison of numeric values is possible instead of statistical evaluation.

The intentions for developing the checklist and the scoring system were similar to those of Wittmers et al. [17] and Aufderheide [16] for defining their soft tissue preservation system, namely to express the degree of soft tissue preservation in a standardized, reproducible format resulting in numerical values. However, the approach and the requirements were different. The above authors used their own system for short description when large numbers of mummies were excavated and for the prediction of internal organ preservation in order to select the best preserved mummies for additional dissection. They state that their estimates for the external examination can be made in less than one minute merely by looking at the body [16]. In contrast, CT examinations require considerable organization and expense and therefore are mostly performed for single mummies or small collections. It follows that a detailed evaluation is essential. One of the strengths of CT is the possibility of soft tissue discrimination, and evaluation of soft tissues is an important component of the complete mummy evaluation. The developed checklist serves as a tool to guide the radiologist through the main soft tissue structures all over the body and enables a standardized checkpoint acquisition with a short to moderate expenditure of time. At the same time the checklist enables a standardized and clear guideline for the assessment of checkpoints as present or absent, instead of requiring extensive descriptions. Assessment of the preservation status of soft tissues is the starting point for detection of possible pathological findings of these tissues. The additional score can easily be calculated by adding the values of all detectable checkpoints. The scoring system allows comparison of mummies by the total score as well as on the level of the two main categories and the various subcategories (S1 Table. Form for documentation of the checkpoints and calculation of the scores). The checklist can be applied to any mummy, irrespective of its method of mummification.

There are some limitations of the study that should be considered. The checklist was developed based upon only two mummy collections. However, these varied considerably in regards to the origin and method of mummification representing a broad variation of preserved soft tissue structures. The selection of checkpoints based upon the experience and focus of the authors, and the scoring system may be subjective to an extent. The definition of checkpoints as “present” or “absent” does not acknowledge the degree of preservation of the different soft tissue structures. The checklist method was chosen in preference to a rating scale because the latter was deemed too multifarious for our aims.

## Conclusions

The developed checklist allows for a standardized and systematic assessment and documentation of soft tissue preservation in whole-body CT examinations of human mummies. The scoring system facilitates a comparison of the soft tissue preservation status between individual mummies and collections in terms of numeric values. We regard the present checklist as an initial version of a “living document” that might be revised following further iterative steps and can be adapted to special demands in future mummy studies.

## Supporting Information

S1 Table Form for documentation of the checkpoints and calculation of the scores. For documentation of the checkpoints rate them as detectable or not detectable (blue shaded fields). For calculation of the scores add the values of detectable checkpoints at the levels of the various subcategories and the two main categories, as well as for the total score (red shaded fields).
(DOC)
