# Supplementary material for: Checklist and Scoring System for the Assessment of Soft Tissue Preservation in CT Examinations of Human Mummies
Source: PLoS One. 2015 Aug 5;10(8):e0133364. doi: 10.1371/journal.pone.0133364 (PMC4526695; doi:10.1371/journal.pone.0133364)
Supplement: S1 Table — For documentation of the checkpoints rate them as detectable or not detectable (blue shaded fields). For calculation of the scores add the values of detectable checkpoints at the levels of the various subcategories and the two main categories, as well as for the total score (red shaded fields). (DOC) [file pone.0133364.s001.doc]

| **NAME OF THE MUMMY:** | | | | | |
| --- | --- | --- | --- | --- | --- |
|  | | | | | |
| **CHECKPOINTS** | **X** | **SCORE** | **CHECKPOINTS** | **X** | **SCORE** |
| **A. Soft Tissues of Head and Musculoskeletal System** |  | **100** | **B. Organs and Organ Systems** |  | **100** |
| **A.1. Head** |  | **20** | **B.1. Central Nervous System and Peripheral Nerves** |  | **10** |
| *nose* |  | 4 | brain *mass/fragments* |  | 1 |
| *auricle* right |  | 2 | **or** |  |  |
| *auricle* left |  | 2 | brain *cerebrum* |  | 2 |
| *ossicles* right |  | 1 | brain *cerebellum* |  | 1 |
| *ossicles* left |  | 1 | brain *brainstem* |  | 1 |
| *bulb and/or lens* right |  | 1 | *trigeminal and/or facial nerve* |  | 1 |
| *optic nerve* right |  | 1 | *spinal cord and/or dura cervical* |  | 1 |
| *eye muscles* right |  | 1 | *spinal cord and/or dura thoracic* |  | 1 |
| *bulb and/or lens* left |  | 1 | *peripheral nerves cervical* |  | 1 |
| *optic nerve* left |  | 1 | *peripheral nerves thoracic* |  | 1 |
| *eye muscles* left |  | 1 | *peripheral nerves lumbar* |  | 0.5 |
| *falx* |  | 2 | *peripheral nerves sacral* |  | 0.5 |
| *tentorium* |  | 2 | **B.2. Cardiorespiratory System** |  | **20** |
| **A.2. Musculoskeletal System** |  | **80** | *hypopharynx and/or larynx* |  | 1 |
| **A.2.1. Tendons and/or Musculature** |  | 40 | *thyroid gland* |  | 5 |
| Neck and Trunk |  |  | *trachea* |  | 2 |
| *skull-base* |  | 4 | *lung* right |  | 2.5 |
| *neck* |  | 4 | *lung* left |  | 2.5 |
| *thoracic and/or lumbar spine* |  | 4 | heart *pericardium* |  | 1 |
| *pelvis* |  | 4 | heart *intraventricular septum* |  | 1 |
| Extremities |  |  | heart *four chambers* |  | 1 |
| *upper arm* right |  | 2 | heart *myocardium* |  | 1 |
| *upper arm* left |  | 2 | heart *valves* |  | 1 |
| *forearm* right |  | 2 | *diaphragm* right |  | 1 |
| *forearm* left |  | 2 | *diaphragm* left |  | 1 |
| *hand* right |  | 2 | **B.3. Gastrointestinal System** |  | **40** |
| *hand* left |  | 2 | *tongue* |  | 5 |
| *thigh* right |  | 2 | *esophagus* |  | 5 |
| *thigh* left |  | 2 | *stomach* |  | 2.5 |
| *lower leg* right |  | 2 | *intestine* |  | 5 |
| *lower leg* left |  | 2 | *rectum and/or anus* |  | 2.5 |
| *foot* right |  | 2 | *liver* |  | 5 |
| *foot* left |  | 2 | *gallbladder* |  | 5 |
| **A.2.2. Peri- and Intra-articular Soft Tissues** |  | 32 | *spleen* |  | 5 |
| shoulder right *rotator cuff* |  | 2 | *pancreas* |  | 5 |
| shoulder right *long biceps tendon* |  | 2 | **B.4. Genitourinary System** |  | **20** |
| shoulder right *capsule and/or labrum* |  | 2 | *kidney* right |  | 2.5 |
| shoulder left *rotator cuff* |  | 2 | *kidney* left |  | 2.5 |
| shoulder left *long biceps tendon* |  | 2 | *urinary bladder* |  | 5 |
| shoulder left *capsule and/or labrum* |  | 2 | *prostate* |  | 5 |
| hip right *capsule and/or labrum* |  | 4 | **or** |  |  |
| hip left *capsule and/or labrum* |  | 4 | *uterus* |  | 5 |
| knee right *anterior cruciate ligament* |  | 1.5 | external genitals *penis* |  | 2.5 |
| knee right *posterior cruciate ligament* |  | 1.5 | external genitals *scrotum* |  | 2.5 |
| knee right *medial meniscus* |  | 1.5 | **or** |  |  |
| knee right *lateral meniscus* |  | 1.5 | external genitals *labia* |  | 5 |
| knee left *anterior cruciate ligament* |  | 1.5 | **B.5. Vasculature-Arteries** |  | **10** |
| knee left *posterior cruciate ligament* |  | 1.5 | *intracranial carotid arteries* |  | 1 |
| knee left *medial meniscus* |  | 1.5 | *cervical carotid arteries* |  | 1 |
| knee left *lateral meniscus* |  | 1.5 | *mediastinal/thoracic arteries* |  | 1 |
| **A.2.3. Intervertebral Discs** |  | 8 | *cornoary arteries* |  | 1 |
| *thoracic spine* |  | 4 | *abdominal aorta* |  | 1 |
| *lumbar spine* |  | 4 | *pelvic arteries* |  | 1 |
|  |  |  | *thigh arteries* right |  | 1 |
|  |  |  | *lower leg arteries* right |  | 1 |
|  |  |  | *thigh arteries* left |  | 1 |
| **Total Score** |  |  | *lower leg arteries* left |  | 1 |

**S1 Table. Form for documentation of the checkpoints and calculation of the scores.**

For documentation of the checkpoints rate them as detectable or not detectable (blue shaded fields).

For calculation of the scores add the values of detectable checkpoints at the levels of the various subcategories and the two main categories, as well as for the total score (red shaded fields).
